# Recurrent stroke shortly after mechanical thrombectomy secondary to carotid web: A case report

**DOI:** 10.1097/MD.0000000000036561

**Published:** 2023-12-15

**Authors:** Guo-Mei Yang, Ren-Wei Zhang, Hua-Gang Li, Yu-Min Liu

**Affiliations:** a Department of Neurology, Zhongnan Hospital of Wuhan University, Wuhan, China.

**Keywords:** acute ischemic stroke, carotid artery stenting, carotid web, mechanical thrombectomy

## Abstract

**Rationale:**

Carotid web, a known source of thrombus for embolic stroke, presents a considerable risk of stroke recurrence. While case reports have demonstrated the safety and effectiveness of mechanical thrombectomy in treating carotid web-related stroke, the need for concurrent carotid artery stenting to prevent recurrent stroke immediately after thrombectomy remains unclear. This study aims to underscore the importance of immediate carotid artery stenting in preventing recurrent stroke following mechanical thrombectomy in patients with carotid web-related stroke.

**Patient concerns:**

A 43-year-old woman with acute onset of left limb weakness and slurred speech within 3 hours was admitted to the emergency department.

**Diagnoses:**

Computed tomographic angiography confirmed the M1 segment occlusion of the right middle cerebral artery.

**Interventions:**

The patient received intravenous thrombolysis in the local hospital and mechanical thrombectomy in our stroke center.

**Outcomes:**

Three days post-mechanical thrombectomy, there was a sudden exacerbation of her neurological deficit symptoms. A reexamination via computed tomographic angiography revealed a re-occlusion in M1 segment of the right middle cerebral artery, despite the implementation of stringent anticoagulation therapy for carotid web-related stroke.

**Lessons:**

Stroke patients with carotid web had a high risk of stroke recurrence and it was necessary to conduct carotid artery stenting to prevent stroke recurrence secondary to the carotid web immediately after mechanical thrombectomy.

## 1. Introduction

The carotid web (CaW) presents as a thin layer of proliferative intimal tissue originating from the arterial wall and extending into the vessel lumen. CaW is commonly found at the beginning of the internal carotid artery or the carotid bulb.^[1]^ Previously classified as an atypical fibromuscular dysplasia, CaW has more recently been considered a separate radiological entity.^[2,3]^ Pathologic features of CaW are characterized by marked thickening of the intima fibroblastic that doesn’t contain the necrotic, cholesterol-rich core of a classic atheroma. In some cases, the fibrous intimal cushion is split by a dissection.^[4]^

CaW is a potential cause of embolic stroke of undermined source. The prevalence of CaW on the same side of stroke ranges from 9.4% to 37% in young patients with cryptogenic stroke.^[5–8]^ Moreover, CaW is associated with a high risk of recurrent stroke/TIA, with varying recurrent times.^[2]^ Diagnosis of carotid web mainly relies on imaging examinations. Computed tomographic angiography (CTA) is the preferred diagnostic method.

Current guidelines for secondary stroke prevention suggest antiplatelet therapy in patients without other etiologies, and invasive treatment involving carotid endarterectomy or carotid artery stenting (CAS) for medically refractory patients.^[9,10]^ However the optimal secondary stroke prevention treatment is unclear. Endovascular treatments are safe and effective in treating CaW-related acute ischemic stroke with large vessel occlusion.^[11–18]^ CAS is usually conducted in the non-acute phase. However, whether it is necessary to conduct carotid artery stenting to prevent stroke recurrence secondary to CaW immediately after mechanical thrombectomy is unknown.

## 2. Case report

**Figure 1.**
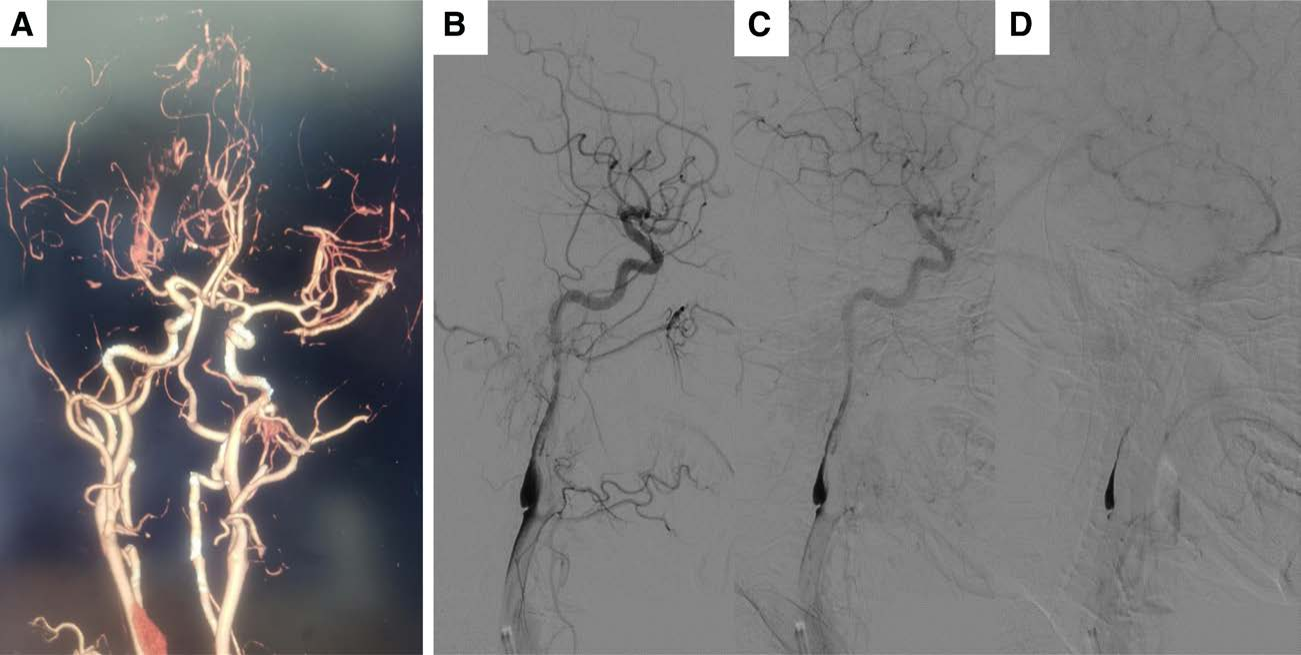
(A) Computed tomography angiography confirmed occlusion of the right middle cerebral artery. (B–D) DSA showed a shelf-like filling defect of the right internal carotid artery with distal contrast stasis. DSA = digital subtraction angiography.

**Figure 2.**
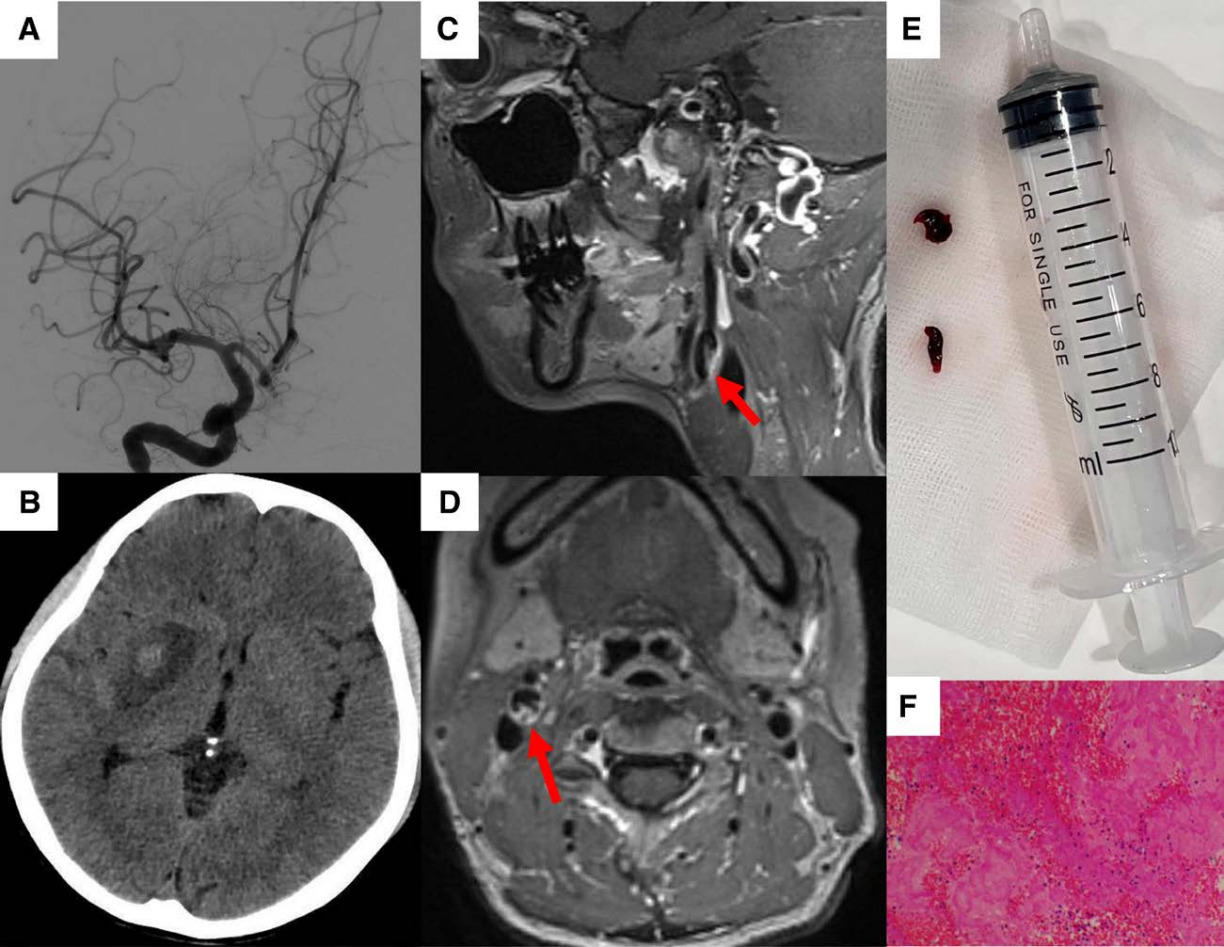
(A) Successful recanalization was achieved after mechanical thrombectomy. (B) Twenty four hours after mechanical thrombectomy, brain CT showed a little intracranial hemorrhage. (C and D) High-resolution MRI showed the carotid web. (E and F) The histological analysis of the thrombus showed a mixed composition of fibrin/platelets and red blood cells. CT = computed tomography.

A 43-year-old woman with acute onset of left limb weakness and slurred speech within 3 hours was admitted to the emergency department in the local hospital. The National Institutes of Health Stroke Scale (NIHSS) score was 12. She did not have a history of diabetes mellitus, hypertension, atrial fibrillation, smoking history, drug abuse, or a family history of early stroke. The blood test and biochemical test were nearly normal. The patient received rt-PA after stroke onset in the local hospital and CTA confirmed occlusion of the right middle cerebral artery (MCA) (Fig. 1A). The patient was transferred to the stroke center of Zhongnan Hospital of Wuhan University for mechanical thrombectomy. The digital subtraction angiography (DSA) showed a shelf-like filling defect of the right internal carotid artery with distal contrast stasis (Fig. 1B–D). Successful recanalization was achieved after mechanical thrombectomy (Fig. 2A). After mechanical thrombectomy, the patient received anticoagulation therapy with Argatroban (10 mg, bid). The patient showed marked improvement in left hemiplegia (NIHSS score 4). 24 hours after mechanical thrombectomy, brain CT showed a little intracranial hemorrhage (Fig. 2B).

To determine the source of embolism, 24 hours long-term electrocardiogram monitoring, transthoracic echocardiography, and transcranial Doppler with bubble test were carried out, but the results were normal. High-resolution MRI showed no arterial stenosis and atherosclerotic plaque in the right middle cerebral artery and showed bilateral internal carotid arteries near the carotid web (Fig. 2C and D). The histological analysis of the thrombus obtained during the mechanical thrombectomy showed a mixed composition of fibrin/platelets and red blood cells (Fig. 2E and F).

**Figure 3.**
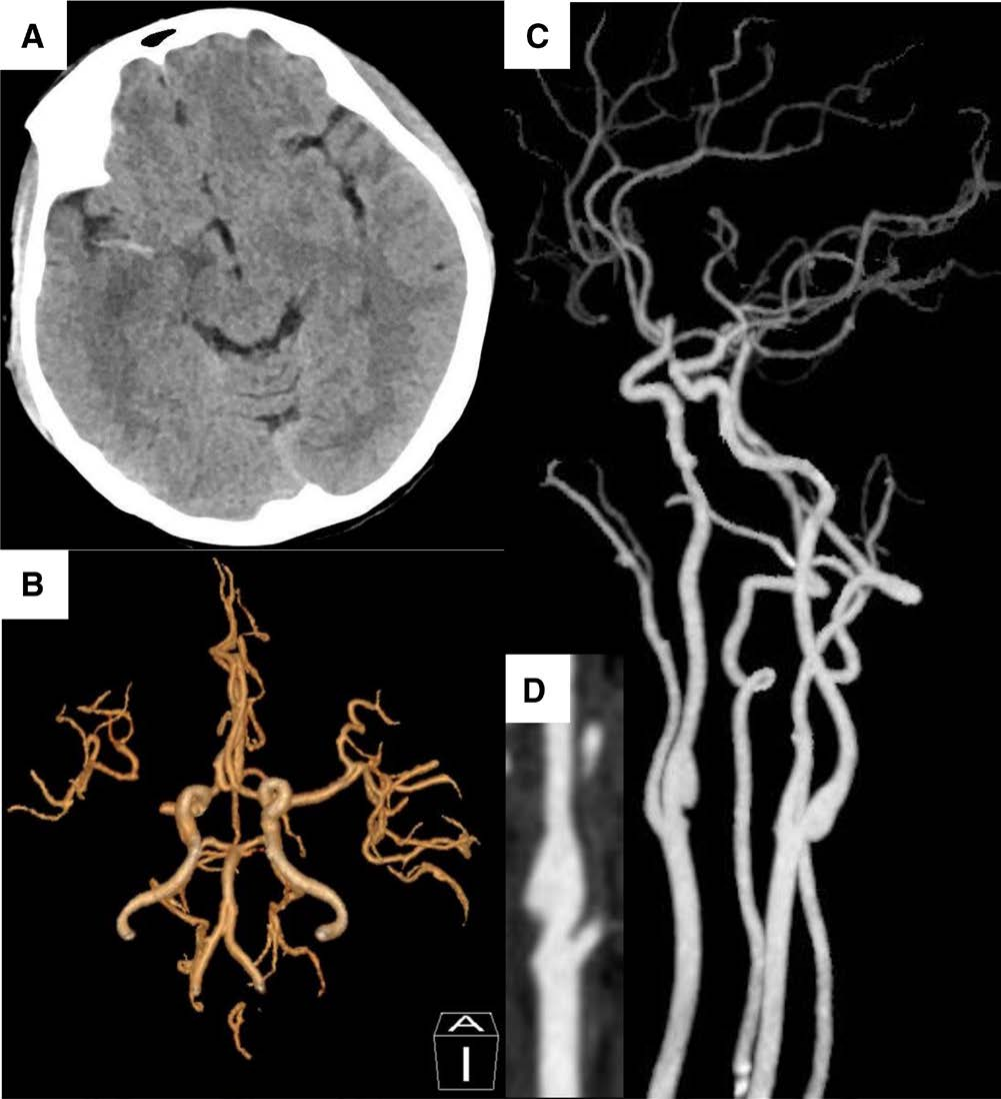
(A) The reexamination of brain CT showed a hyperdense MCA sign and (B) computed tomographic angiography showed M1 of right middle cerebral artery re-occluded and (C and D) the thrombus superimposed to the web. CT = computed tomography, MCA = middle cerebral artery.

Three days after the mechanical thrombectomy, the patient’s neurological deficit symptoms suddenly got worse (NIHSS score 9) and a reexamination of brain CT showed a hyperdense MCA sign (Fig. 3A) and CTA showed M1 of right middle cerebral artery re-occluded (Fig. 3B) and the thrombus superimposed to the web (Fig. 3C and D). Due to the high risk of hemorrhagic complications, the patient did not undergo mechanical thrombectomy again.

Two weeks later, the patient was discharged to another hospital for rehabilitation with residual left extremity weakness with Manual Muscle Testing scores 1/5 and NIHSS score 6. At 2-month follow-up, repeated CTA revealed the resolution of the superimposed thrombus and the presence of a typical CaW, and the middle cerebral artery continued to be occluded with a Modified Rankin Scale score of 2.

## 3. Discussion

Stroke patients with CaW had a high risk of stroke recurrence. The recurrence rate of ipsilateral stroke/TIA in stroke patients with the CaW was 17% to 40%.^[11,12,19–22]^ Choi et al reported 5 of 7 (71.4%) stroke patients with CaW experienced recurrent stroke, and the time of recurrence ranged from 1 to 97 months during an 8-year follow-up.^[4]^ This is significantly higher than the 5% per year recurrent rate for cryptogenic ischemic stroke and the 3% recurrent rate for large vessel occlusive stroke without CaW.^[19,23]^ The time of recurrent stroke varied from hours to years in the literature. and it was short in cryptogenic stroke patients with CaW. During a median follow-up period of 12.2 months, 7 patients (32%) developed recurrent stroke/TIA involving previously symptomatic CaW areas with a median time to event of 13 months, 3 at 1 week, 2 at 1 year, 1 case occurred within 24 hours after thrombolysis treatment.^[11]^ In our case, the patient suffered ipsilateral middle cerebral artery occlusion 3 days after mechanical thrombectomy.

The high risk of ipsilateral stroke and stroke recurrence in patients with CaW may be related to morphologic characteristics of CaW and corresponding hemodynamic changes. The underlying mechanism of CaW-related stroke may be a series of processes, blood turbulence, and blood flow stagnation, leading to thrombus formation, thrombus shedding, and cerebral embolism, which lead to stroke/TIA.^[8]^ In our patient, we observed a delayed clearance of contrast agent at the distal end of the CaW during DSA (Fig. 1B–D), and the thrombus formation on the distal portion of the CaW on CTA (Fig. 3D). The removed embolus during intervention was found to be a mixed thrombus, consistent with findings reported in other literature.^[4,24]^ Moreover, comparing histological features of clot composition in situ and secondary cerebrovascular embolized thrombi caused by CaW, it was found that the in situ thrombus showed mainly fibrin, and the embolized thrombus had the same content of red blood cells and fibrin/platelets.^[25,26]^ Computational fluid dynamics studies using artificial models of CaW had shown blood flow disturbances. Studies have shown that thrombus formation at the CaW is associated with changes in the hemodynamic pattern. Specifically, factors such as decreased blood flow velocity, formation of turbulence, increased recirculation of blood, and elevated shear stress within the vessel lumen collectively contribute to an increased risk of thrombus formation, thereby increasing the risk of ischemic stroke.^[4,27–29]^

There are several useful tools to identify CaW. DSA has been considered the gold standard for the diagnosis of CaW, with a shelf-like filling defect and contrast agent retention until the venous phase. However, standard posteroanterior or lateral projections may miss CaW due to being located in the posterior wall of the carotid artery. In contrast, computed tomographic angiography (CTA) has the advantages of multiplanar reconstruction and high sensitivity and is the preferred diagnostic method. The CaW is shown as a thin intraluminal filling defect along the posterior wall of the carotid bulb beyond the carotid bifurcation on oblique sagittal section and as a septum on axial section.^[4]^ Doppler ultrasonography (DUS) could simultaneously evaluate carotid artery luminal, wall, and blood flow. On routine ultrasound, the CaW presents as an isoechoic or hypoechoic filmlike structure, and with “cliff-like” arterial stenosis in the longitudinal section. On color DUS, there are swirl blood flow and pooling of blood with contrast stagnation distal to the lesion.^[1,2]^ However, DUS likely has lower sensitivity for diagnosing CaW and overestimates the degree of stenosis compared with CTA. High-resolution MRI is also used in recent years, but the performance is inconsistent in previous publications.^[30,31]^ For an accurate diagnosis, it is necessary to rely on multimodal imaging rather than a single image. In addition, CaW is often identifiable through imaging, but it can be mistaken for other conditions such as small protruding lesions, atherosclerotic plaques, and arterial dissection. It is necessary to fully evaluate the clinical information to avoid misdiagnosis.

The stroke mechanism in CaW patients is embolism. In theory, antithrombogenic therapy, especially anticoagulation, should be an effective choice for secondary stroke prevention. The 2021 AHA/ASA guideline recommended medical management with antithrombogenic therapy as first-line treatment. However, medical management is debated. Many studies have uncovered that a considerable proportion of patients undergoing medical therapy still suffer from recurrent stroke. In certain cases, stroke may recur even while they are taking the medication. In a systematic review by Zhang et al, 56% of patients with symptomatic CaW receiving medical treatment had recurrent stroke.^[18]^ In the MR CLEAN study, the 2-year risk of recurrent stroke in treated stroke patients with CaW was 5/25 (20%). In comparison, CAS or carotid endarterectomy seems to solve the problem at its root-eliminating cul-de-sacs and avoiding turbulence and thrombus formation.^[22]^ Since the first report of CAS as secondary stroke prevention in CaW patients in 2014,^[3]^ Many studies about CAS for CaW-related stroke have been published in literature.^[11–18]^ Only a few patients who underwent stent placement in non-acute phase experienced short-term post-stenting bradycardia or hypotension.^[32]^ No ischemic or hemorrhagic procedural complications were observed, and no patients had recurrent stroke or transient ischemic attack (TIA) during the follow-up period. A study prospectively and consecutively enrolled patients < 65 years old with cryptogenic stroke identified 24 patients with CaW, 16 patients underwent stenting at a median of 12.2 (7.0–18.7) days after stroke with no periocedural complications. No recurrence of stroke/TIA occurred in individuals (median follow-up 4 [2.4–12.0] months).^[11]^ In a systematic review, Of the 135 symptomatic CaW, 35 had carotid artery stenting. All patients had no procedural complications and remained stroke-free during follow-up (median duration 10.7 months, range 3–144 months).^[18]^ In conclusion, CAS is a safe and effective treatment for symptomatic CaW.

The timing of stent placement is unclear, mostly in the next days or months after stroke/TIA in previous reports. Stent placement should be performed as soon as possible because of the high proportion of recurrence in a short time after stroke. We evaluated published literature on CAS. Only two patients underwent stenting while mechanical thrombectomy. A first-time stroke patient with CaW identified by CTA underwent emergent mechanical thrombectomy and stent placement after administered tPA. The patient showed marked improvement with no residual deficit or recurrent symptoms. At 4-month follow-up, conventional angiogram showed patent stent without in-stent stenosis. In another case, the patient’s MCA was reoccluded the day after mechanical thrombectomy. The patient underwent repeat thrombectomy and carotid stent placement.^[17,33]^ Therefore, after a comprehensive evaluation, CAS can be performed concurrently with mechanical thrombectomy, allowing for immediate addressing of the underlying cause.

This case report offers some insights into the question of whether simultaneous stent implantation is necessary during mechanical thrombectomy for patients with rare CaW-related stroke. However, there are some limitations. Firstly, this is a retrospective case report, possibly susceptible to inherent bias. A substantial prospective study with large sample size is imperative to conclusively validate the efficacy and safety of stent implantation following mechanical thrombectomy in CaW-related stroke. It is based on retrospective data from a single case, lacks a control group, and cannot establish a causal relationship between stent implantation and recurrence risk. Additionally, findings from this single case may not be broadly applicable. Moreover, the patient in this case experienced symptomatic intracranial hemorrhage after mechanical thrombectomy, which might affect the preventive effectiveness of stent implantation. Assessing bleeding risk and its contributing factors will be a key focus for future research. Thus, prospective clinical trials with an adequate sample size are needed to comprehensively evaluate bleeding risks associated with interventional treatments and determine the optimal timing for stent placement.

## 4. Conclusion

Stroke patients with CaW had a high recurrent rate and early recurrence time, so early intervention is required. The recurrence risk is related to the hemodynamic derangement caused by CaW. Carotid artery stenting is safe and effective and can solve the problem at its root. In the case of full assessment, stent placement while mechanical thrombectomy may be a better choice for treating CaW. Further studies are needed to determine if it is an effective strategy for reducing stroke recurrence rates.

## Author contributions

**Data curation:** Ren-Wei Zhang, Hua-Gang Li.

**Methodology:** Guo-Mei Yang, Ren-Wei Zhang.

**Resources:** Ren-Wei Zhang, Hua-Gang Li.

**Supervision:** Yu-Min Liu.

**Writing – original draft:** Guo-Mei Yang.

**Writing – review & editing:** Ren-Wei Zhang, Yu-Min Liu.

## References

[1] Liang SX Qin PX Xie LL. The carotid web: Current research status and imaging features. Front Neurosci. 2023;17:1104212. 36860618 10.3389/fnins.2023.1104212 PMC9968728

[2] Olindo S Marnat G Chausson N. Carotid webs associated with ischemic stroke Updated general review and research directions. Rev Neurol (Paris). 2021;177:627–38. 33455831 10.1016/j.neurol.2020.09.007

[3] Lenck S Labeyrie MA Saint-Maurice JP. Diaphragms of the carotid and vertebral arteries: an underdiagnosed cause of ischaemic stroke. Eur J Neurol. 2014;21:586–93. 24447601 10.1111/ene.12343

[4] Choi PMC Singh D Trivedi A. Carotid webs and recurrent ischemic strokes in the era of CT angiography. AJNR Am J Neuroradiol. 2015;36:2134–9. 26228877 10.3174/ajnr.A4431 PMC7964886

[5] Joux J Boulanger M Jeannin S. Association Between carotid bulb diaphragm and ischemic stroke in young afro-caribbean patients: a Population-Based Case-Control Study. Stroke. 2016;47:2641–4. 27625379 10.1161/STROKEAHA.116.013918

[6] Coutinho JM Derkatch S Potvin ARJ. Carotid artery web and ischemic stroke A case-control study. Neurology. 2017;88:65–9. 27864523 10.1212/WNL.0000000000003464 PMC5200857

[7] Sajedi PI Gonzalez JN Cronin CA. Carotid bulb webs as a cause of “cryptogenic” ischemic stroke. AJNR Am J Neuroradiol. 2017;38:1399–404. 28495950 10.3174/ajnr.A5208 PMC7959897

[8] Kim SJ Nogueira RG Haussen DC. Current understanding and gaps in research of carotid webs in ischemic strokes a review. JAMA Neurol. 2019;76:355–61. 30398546 10.1001/jamaneurol.2018.3366

[9] Kleindorfer DO Towfighi A Chaturvedi S. 2021 Guideline for the prevention of stroke in patients with stroke and transient ischemic attack: a Guideline From the American Heart Association/American Stroke Association. Stroke. 2021;52:e364–467. 34024117 10.1161/STR.0000000000000375

[10] Chinese Society of Neurology CSS. Chinese guideline for the secondary prevention of ischemic stroke and transient ischemic attack 2022. Chin J Neurol. 2022;55:1071–110.

[11] Haussen DC Grossberg JA Bouslama M. Carotid web (intimal fibromuscular dysplasia) has high stroke recurrence risk and is amenable to stenting. Stroke. 2017;48:3134–7. 29018133 10.1161/STROKEAHA.117.019020

[12] Haussen DC Grossberg JA Koch S. Multicenter experience with stenting for symptomatic carotid web. Interv Neurol. 2018;7:413–8. 30410519 10.1159/000489710 PMC6216720

[13] Mathew S Davidson DD Tejada J. Safety and feasibility of carotid revascularization in patients with cerebral embolic strokes associated with carotid webs and histopathology revisited. Interv Neuroradiol. 2021;27:235–40. 33322975 10.1177/1591019920980271 PMC8050519

[14] Pereira BJA Batista UC Tosello RT. Web vessels: literature review and neurointerventional management. World Neurosurg. 2018;110:e907–16. 29191528 10.1016/j.wneu.2017.11.115

[15] Patel SD Otite FO Topiwala K. Interventional compared with medical management of symptomatic carotid web: A systematic review. J Stroke Cerebrovasc Dis. 2022;31:106682. 35998383 10.1016/j.jstrokecerebrovasdis.2022.106682

[16] Martinez-Perez R Lownie SP Pandey SK. Stent placement for carotid web. World Neurosurg. 2017;98:879.e9–879.e11. 10.1016/j.wneu.2016.11.050 27876658

[17] Wojcik K Milburn J Vidal G. Carotid webs: radiographic appearance and significance. Ochsner J. 2018;18:115–20. 30258290 10.31486/toj.18.0001 PMC6135290

[18] Zhang AJ Dhruv P Choi P. A systematic literature review of patients with carotid web and acute ischemic stroke. Stroke. 2018;49:2872–6. 30571430 10.1161/STROKEAHA.118.021907

[19] Guglielmi V Compagne KCJ Sarrami AH. Assessment of recurrent stroke risk in patients with a carotid web. JAMA Neurol. 2021;78:826–33. 33970205 10.1001/jamaneurol.2021.1101 PMC8111564

[20] Joux J Chausson N Jeannin S. Carotid-bulb atypical fibromuscular dysplasia in young afro-caribbean patients with stroke. Stroke. 2014;45:3711–3. 25358695 10.1161/STROKEAHA.114.007313

[21] Sajedi P Chelala L Nunez-Gonalez J. Carotid webs and ischemic stroke: Experiences in a comprehensive stroke center. J Neuroradiol. 2019;46:136–40. 30273631 10.1016/j.neurad.2018.09.003

[22] Olindo S Chausson N Signate A. Stroke recurrence in first-ever symptomatic carotid web: a Cohort Study. J Stroke. 2021;23:253–62. 34102760 10.5853/jos.2020.05225 PMC8189848

[23] Hart RG Sharma M Mundl H. Rivaroxaban for stroke prevention after embolic stroke of undetermined source. N Engl J Med. 2018;378:2191–201. 29766772 10.1056/NEJMoa1802686

[24] Rodriguez-Castro E Arias-Rivas S Santamaria-Cadavid M. Carotid web: the challenging diagnosis of an under-recognized entity. J Neurol. 2022;269:5629–37. 35713691 10.1007/s00415-022-11210-y

[25] Gao Q Hu S Yang XM. Histologic differences between in situ and embolized carotid web thrombi: a case report. BMC Neurol. 2021;21:1–5. 34645398 10.1186/s12883-021-02428-w PMC8513242

[26] Haynes J Raz E Tanweer O. Endarterectomy for symptomatic internal carotid artery web. J Neurosurg. 2021;135:1–8. 32858515 10.3171/2020.5.JNS201107

[27] Compagne KCJ Dilba K Postema EJ. Flow patterns in carotid webs: a Patient-Based Computational Fluid Dynamics Study. AJNR Am J Neuroradiol. 2019;40:703–8. 30872422 10.3174/ajnr.A6012 PMC7048501

[28] Ozaki D Endo T Suzuki H. Carotid web leads to new thrombus formation: computational fluid dynamic analysis coupled with histological evidence. Acta Neurochir. 2020;162:2583–8. 32152755 10.1007/s00701-020-04272-2

[29] Bae T Ko JH Chung J. Turbulence intensity as an indicator for ischemic stroke in the carotid web. World Neurosurg. 2021;154:e443–57. 34325025 10.1016/j.wneu.2021.07.049

[30] Fushimi Y Yoshida K Okawa M. Vessel wall MR imaging in neuroradiology. Radiol Med. 2022;127:1032–45. 35907157 10.1007/s11547-022-01528-y PMC9362557

[31] Zhu CT Li ZX Ju Y. Detection of carotid webs by CT angiography, high-resolution MRI, and ultrasound. J Neuroimaging. 2021;31:71–5. 32986890 10.1111/jon.12784

[32] Brinjikji W Agid R Pereira VM. Carotid stenting for treatment of symptomatic carotid webs: a single-center case series. Interv Neurol. 2018;7:233–40. 29765392 10.1159/000486537 PMC5939846

[33] Mac Grory B Emmer BJ Roosendaal SD. Carotid web: an occult mechanism of embolic stroke. J Neurol Neurosurg Psychiatry. 2020;91:1283–9. 33004431 10.1136/jnnp-2020-323938

